# Coenzyme Q10 and Silymarin Reduce CCl_4_-Induced Oxidative Stress and Liver and Kidney Injury in Ovariectomized Rats—Implications for Protective Therapy in Chronic Liver and Kidney Diseases

**DOI:** 10.3390/pathophysiology28010005

**Published:** 2021-01-18

**Authors:** Samanta Sifat Lamia, Tushar Emran, Jubaida Khatun Rikta, Nowreen Islam Chowdhury, Manoneeta Sarker, Preeti Jain, Tabinda Islam, Zarin Tasnim Gias, Manik Chandra Shill, Hasan Mahmud Reza

**Affiliations:** Department of Pharmaceutical Sciences, School of Health and Life Sciences, North South University, Bashundhara R/A, Dhaka 1229, Bangladesh; samanta.sifat@northsouth.edu (S.S.L.); tushar.emran@northsouth.edu (T.E.); jubaida.khatun@northsouth.edu (J.K.R.); nowreen.chowdhury@northsouth.edu (N.I.C.); manonees95@zedat.fu-berlin.de (M.S.); preti.jain@northsouth.edu (P.J.); tabinda.islam@northsouth.edu (T.I.); zarin.gias@northsouth.edu (Z.T.G.); manik.shill@northsouth.edu (M.C.S.)

**Keywords:** coenzyme Q10, CCl_4_, silymarin, ovariectomized rat model, oxidative stress

## Abstract

Oxidative stress is one of the key factors in the pathophysiology of liver disease. The present study aimed to evaluate the potential impact of two antioxidants, namely coenzyme Q10 (CoQ10) and silymarin, on carbon tetrachloride (CCl_4_)-induced oxidative stress and hepatic damage in ovariectomized rats. Female Long Evans rats were divided into six groups (n = 6): control, CCl_4_, CCl_4_ + CoQ10 (200 mg/kg), CCl_4_ + silymarin (140 mg/kg), Control + CoQ10, and Control + silymarin. Plasma and tissues from liver and kidney were analyzed for oxidative stress parameters and antioxidant enzyme activities using biochemical assays. Infiltration of inflammatory cells and fibrosis were assessed by histological staining of tissue sections. Both CoQ10 and silymarin significantly lowered serum alanine aminotransferase (ALT), aspartate aminotransferase (AST), and alkaline phosphatase (ALP) levels that were detected to be higher in CCl_4_ rats compared to controls. Significant reduction in CCl_4_-induced elevated levels of oxidative stress markers malondialdehyde (MDA), nitric oxide (NO), and advanced protein oxidation product (APOP) was observed with both antioxidants. However, in control rats, CoQ10 and silymarin did not produce a significant effect. Histological analysis revealed that CCl_4_ markedly increased the level of inflammatory cells infiltration and fibrosis in liver and kidney tissues, but this was significantly reduced in CCl_4_ + CoQ10 and CCl_4_ + silymarin groups. Taken together, our results suggest that CoQ10 and silymarin can protect the liver against oxidative damage through improved antioxidant enzyme activities and reduced lipid peroxidation. Thus, supplementation of the aforementioned antioxidants may be useful as a therapeutic intervention to protect liver health in chronic liver diseases.

## 1. Introduction

Excessive production of free radicals, such as superoxide, hydroxyl, hydrogen peroxide, and nitric oxide radicals in living beings causes homeostatic imbalance and oxidative stress leading to damage to biological molecules such as proteins, nucleic acids, and membrane lipids [1]. Alteration in cellular constituents and level of antioxidant enzymes such as superoxide dismutase (SOD), catalase (CAT), and reduced glutathione (GSH) due to increased oxidative stress results in chronic kidney disease (CKD) and liver disease, which are among the major causes of morbidity and mortality worldwide and raise serious public health concerns [1,2,3,4]. Pathogenesis of liver diseases such as chronic hepatitis, fibrosis, fatty liver, cirrhosis, and hepatocellular carcinoma has been reported to be associated with oxidative stress in several studies [4]. Carbon tetrachloride (CCl_4_) is a well-known hepatotoxin used for studying hepatotoxicity leading to pathogenesis of hepatic damage in animal models [5]. Metabolic products of CCl_4_ lead to the generation of trichloromethyl and proxy chloromethyl free radicals, causing an imbalance between reactive oxygen species (ROS) and antioxidants and resulting in liver damage and necrosis. CCl_4_ can also activate Kupffer cells by enhancing Ca^2+^ concentration, initiating the intracellular release of harmful cytokines that facilitates the death of hepatocytes and generation of oxidative stress [6]. Ovariectomized rat models are often used in clinical studies to overcome the antioxidant effects of estrogen, an ovarian hormone that can inhibit lipid peroxidation and reduce lipoprotein oxidation and superoxide anion production [1,7]. In liver diseases, estrogen also plays a protective role through mechanisms such as inhibition of fibrogenesis, cellular senescence, increase in innate immunity, and protection of mitochondrial structure and function [8].

Coenzyme Q10 is an intracellular antioxidant that protects the cells by preventing lipid peroxidation and the regeneration of other antioxidants that prevent free-radical-induced oxidative damage [9]. CoQ10 can withstand oxidation-reduction cycles, and the concentration of CoQ10 is higher in organs such as kidneys, heart, and liver, which demonstrate high metabolic rate and need coenzyme Q10 as a sufficient energy support system [10]. Chemically, coenzyme Q10 is a lipid-soluble benzoquinone that functions as a diffusible electron carrier in the mitochondrial respiratory chain, thereby scavenges free radicals efficiently [10]. The protective role of CoQ10 has also been reported in oxidative and inflammatory tissue damage [9]. CoQ10 prohibits the reduction in GSH level and catalase activity and exhibits anti-inflammatory properties by decreasing the production of pro-inflammatory cytokines such as tumor necrosis factor [11]. Diminished coenzyme Q10 level may be attributed to nutritional deficiencies such as vitamin B6 deficiency, genetic defects, and poor synthesis and utilization of CoQ10 [10]. Again, damaged, sick, or aging tissues demand the enzyme to a higher extent than normal because of increased oxidative stress, and drugs such as antidepressants, beta-blockers, and cholesterol-lowering agents also reduce the level of CoQ10 [10,11].

Silymarin is a naturally occurring polyphenolic compound isolated from *Silybum marianum* and known to possess strong antioxidant, anti-inflammatory, and anti-fibrotic properties [12]. Role of silymarin in hepatoprotection and neuroprotection has been reported in several studies [13]. Being potent antioxidants, both CoQ10 and silymarin have gained much attention of researchers due to their possible therapeutic effects in oxidative stress associated disorders such as cancer, diabetes, neurodegenerative, hepatic, and cardiovascular diseases [14]. In addition, exogeneous supplementation of these antioxidants has been reported to be safe in humans with minimal side effects [14].

Against this background, the current study aimed to evaluate the ameliorative effect of CoQ10 and silymarin on CCl_4_-induced liver and kidney damage including oxidative stress in ovariectomized rats. Use of the ovariectomized rat model is unique in this study and will help in assessing the precise and unbiased role of CoQ10 and silymarin in restoring CCl_4_-induced liver and kidney damage without being influenced by ovarian hormones that are known to exhibit protective effects in pathologies related to oxidative stress [15,16,17].

## 2. Materials and Methods

### 2.1. Experimental Animals and Care

Forty female Long Evans rats (240 ± 5 g) were collected from the Animal House of the Department of Pharmaceutical Sciences, North South University. To be eligible for inclusion in the experiments, rats were checked for certain parameters such as weight and fitness. Also, to avoid hormonal as well as sexual interference, selected rats were kept separated from the male rats following their birth. The animals were kept in individual cages at a room temperature of 20–26 °C under a 12 h light/12 h dark cycle. Free access to a standard pelleted diet (20 g per rat) and water was ensured.

### 2.2. Study Design and Treatment

Animals were anesthetized by injecting ketamine hydrochloride 100 mg/kg through the intraperitoneal route. A 2 cm long midline dorsal incision was made in the lower back of the rat just below the bottom of rib cage to access the ovaries. A single ligature around the oviduct was made to prevent bleeding due to the removal of the ovaries. After careful removal of the ovaries, the cut was finally closed using a 4-O sterile suture. All the animals were kept under close observation for 7 days. During the 1-week post-surgery observational period, four rats were discarded based on weight and post-surgery trauma. The remaining 36 rats were divided into six groups, each consisting of six rats. The groups were treated as follows: Group I (Control), Group II (CCl_4_), Group III (CCl_4_ + CoQ10), Group IV (CCl_4_ + silymarin), Group V (Control + CoQ10), and Group VI (Control + silymarin). Rats were orally administered with CoQ10 at a dose of 200 mg/kg/day body weight, silymarin at 140 mg/kg/day body weight, and CCl_4_ at a dose of 1 mL/kg body weight twice a week for 4 weeks [18,19]. Body weight and food and water intake were monitored regularly. At the end of experimentation period, all animals were sacrificed and liver, kidney, and blood samples were collected. The organs were weighed immediately and were preserved in buffered formalin solution (pH 7.4) for histopathology. For the bioassay, collected liver and kidney samples were sonicated and centrifuged with phosphate-buffered saline (PBS), and the supernatants were collected for further assay. The blood samples were centrifuged immediately at 12,000 rpm for 10 min at 4 °C and stored in microcentrifuge tubes at −20 °C for further analysis.

### 2.3. Biochemical Assays

#### 2.3.1. Measurement of 17β-Estradiol

Whole blood was collected from ovariectomized and non-ovariectomized rats after 2 weeks following recovery and subjected to centrifugation to separate serum. Each serum was divided into 25 μL aliquots and stored at −20 °C. Later, 17β-estradiol was measured using an electrochemiluminescence immunoassay kit (Roche diagnostics, USA) as per manufacturer’s instruction.

#### 2.3.2. Estimation of Lipid Peroxidation as Malondialdehyde (MDA)

Lipid peroxidation was estimated colorimetrically, with thiobarbituric acid reactive substances (TBARS) indicating the lipid peroxidation and oxidative stress as previously described [20]. The result was expressed as nm of MDA per mg protein.

#### 2.3.3. Estimation of Nitric Oxide (NO)

NO was determined according to the protocol as previously described [21] with some modifications. Briefly, 60 μL tissue homogenate or plasma samples were taken in a 96-well plate and mixed with 1% sulfanilamide and 0.1% N-(1-naphthyl) ethylenediamine hydrochloride (NED) in 2.5% H_3_PO_4_. After 20 min incubation at room temperature, a pink chromophore was generated due to diazotization of nitrite ions with sulfanilamide and subsequent coupling with NED. Finally, the absorbance was recorded at 540 nm against a blank sample. A standard curve was made by serial dilution of 1 mM sodium nitrite stock solution.

#### 2.3.4. Determination of Advanced Protein Oxidation Products (APOP)

APOP levels were determined by a previously used method as described [22]. Tissue/plasma was taken with PBS at a ratio of 1:5 with 0.1 mL of KI. After 2 min, acetic acid was added, and absorbance was measured at 340 nm.

#### 2.3.5. Determination of GSH

The reduced level of glutathione was estimated using the method previously described [23]. Briefly, 10 µL tissue homogenate/plasma and 90 µL PBS were taken in an eppendorf tube. Then, 100 µL DTNB (5,5-dithiobis-2-nitrobenzoic acid) was added and incubated for an hour at 4 °C. After formation of yellow color, the absorbance was measured at 412 nm.

#### 2.3.6. Determination of CAT

CAT activities were determined using a method previously described [24]. Briefly, 10 µL tissue/plasma and 90 µL PBS were taken in an Eppendorf tube, and 40 µL H_2_O_2_ was added. The absorbance was taken at 240 nm. Changes in the absorbance of the reaction solution at 240 nm were determined after 1 min. One unit of CAT activity was defined as a change in the absorbance of 0.01 unit/min.

#### 2.3.7. Determination of SOD Level

The activity of superoxide dismutase enzyme was assayed using a modified procedure described previously [25]. Briefly, 10 µL (tissue/plasma) and 90 µL PBS were taken. Then, 100 µL adrenaline was added, and the absorbance was measured at 480 nm. SOD activity was determined in terms of its ability to inhibit autooxidation of adrenaline to adrenochrome. A control reaction consisting of all the ingredients except the tissue/plasma was analyzed simultaneously.

#### 2.3.8. Assessment of Liver Toxicity

Blood samples were collected and centrifuged at 10,000 rpm for 15 min at 4 °C. Plasma was separated, transferred to 1.5 mL Eppendorf tubes, and stored at −20 °C until further analysis was conducted. Enzyme activities of aspartate transaminase (AST), alanine transaminase (ALT), and alkaline phosphatase (ALP) were determined using specific kits (Human Diagnostics, Germany) according to the manufacturer’s protocols.

### 2.4. Histopathology Procedure

Following overnight fixation in 10% formalin, the tissue blocks were dehydrated in ascending concentration of ethyl alcohol. Tissues were then treated in xylene, embedded in paraffin, and subjected to sectioning at a thickness of 5 micron. The slides containing sections were deparaffinized with xylene, followed by rehydration of tissues in alcohol of different concentrations from high to low. Later, tissue sections were stained with hematoxylin and sirius red. Finally, stained slides were cleaned in xylene, mounted, and observed under a light microscope (Olympus DP12, Japan) at 20× magnification.

### 2.5. Statistical Analysis

Data obtained in this study were analyzed using SigmaPlot 14.0. Analysis of variance (ANOVA) with Newman-Keuls post-hoc test was performed. Differences were considered statistically significant at *p* ≤ 0.05.

## 3. Results

### 3.1. Effect of CoQ10 Supplementation on Body Weight of Ovariectomized Rats

**Figure 1:**
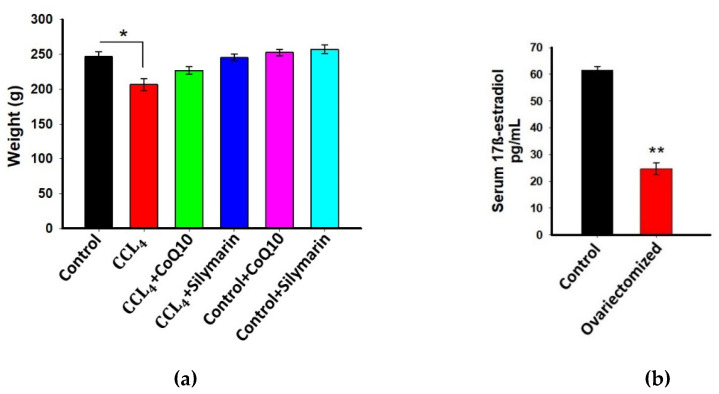
Effect of coenzyme Q10 (CoQ10) and silymarin on weight of ovariectomized female rats (**a**) Levels of 17β-estradiol in serum of ovariectomized and non-ovariectomized rats (control); (**b**) Data are presented as mean ± SD, n = 6. Statistical analysis involved post-hoc tests. Statistical significance is indicated as * *p* ≤ 0.05 and ** *p* ≤ 0.01.

The average body weight of the experimental rats was determined before the start of drug intervention and was 240 ± 5 g. During the entire experimentation period, all rats were weighed routinely, and we found no substantial changes in body weight between the different groups except the rats that received only CCl_4_ (Figure 1a). Notably, in the last week of treatment, there was marked reduction in body weight in CCl_4_-treated rats as compared to other groups. We further observed that administration of CoQ10 or silymarin had no relation with weight loss or weight gain compared to control rats.

### 3.2. Estimation of 17β-Estradiol in Plasma

Previous studies demonstrated that estrogens have a protective effect that is accomplished through their antioxidant, anti-inflammatory, and anti-fibrogenic actions [1,7]. Therefore, in this study, we used ovariectomized rats to avoid the interference of estrogens and to assess the actual effect of CoQ10 and silymarin. To confirm that ovariectomy essentially reduced 17β-estradiol level in ovariectomized rats, blood serum was collected from both ovariectomized and non-ovariectomized (control) rats, and the biologically most active form of estrogen, 17β-estradiol was measured. As expected, we found a significant reduction of 17β-estradiol in ovariectomized rats as compared to controls (*p* ≤ 0.01) (Figure 1b).

### 3.3. Effect of CoQ10 Supplementation on Oxidative Stress Level in Kidney, Liver, and Plasma

**Figure 2:**
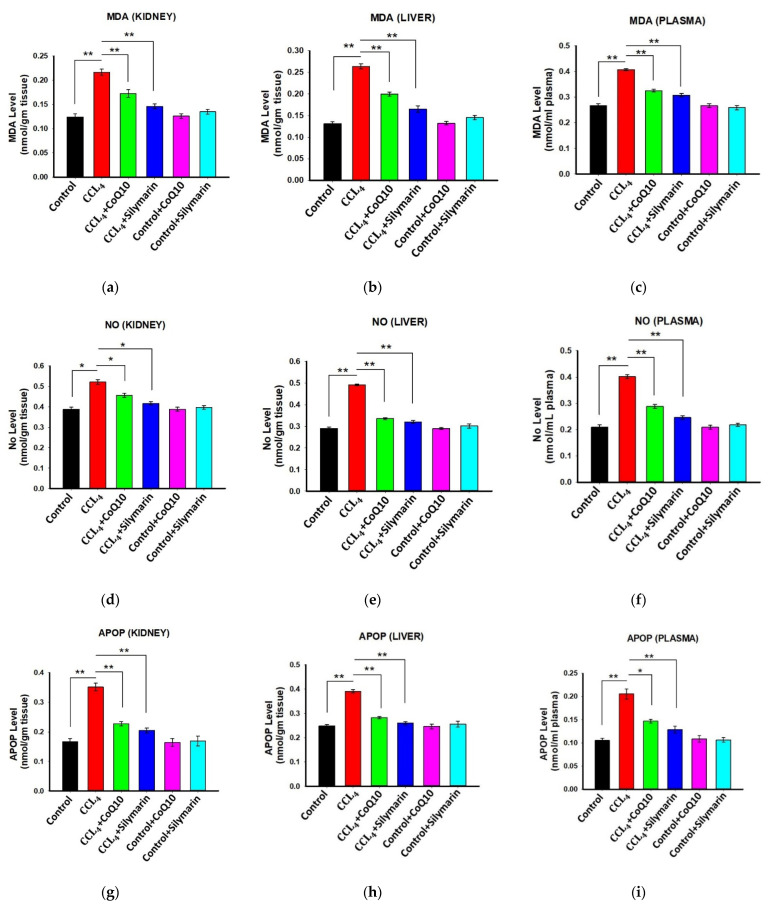
Effect of CoQ10 and silymarin on MDA (**a**–**c**), NO (**d**–**f**), and advanced protein oxidation product (APOP) (**g**–**i**) levels in kidney, liver, and plasma of ovariectomized female rats. Data are presented as mean ± SD, n = 6, where n = 6. Statistical analysis involved post-hoc tests. Statistical significance is indicated as * *p* ≤ 0.05 and ** *p* ≤ 0.01.

In the experimental rats, we first confirmed the level of oxidative stress by measuring several stress markers before administration of CoQ10 or silymarin. In CCl_4_ rats, significant elevation of malondialdehyde (MDA) levels in kidney, liver, and plasma was observed as compared to the control group (*p* ≤ 0.01) (Figure 2a–c). However, treatment with CoQ10 and silymarin reduced MDA levels markedly in these tissues and plasma (*p* ≤ 0.01). No significant change in MDA level was observed in control rats when they were administered either CoQ10 or silymarin. Also, the results obtained with either CoQ10 or silymarin were not statistically different.

Similarly, NO levels (Figure 2d–f) in kidney, liver, and plasma of CCl_4_ rats were higher compared to the control, and they were significantly smaller in the CCl_4_ + CoQ10 (*p* ≤ 0.05 in kidney; *p* ≤ 0.01 in liver and plasma) and CCl_4_ + silymarin (*p* ≤ 0.05 in kidney; *p* ≤ 0.01 in liver and plasma) groups.

We further analyzed the effect of CoQ10 and silymarin on the APOP level in different groups. The CCl_4_ rats exhibited increased levels of APOP in the kidney, liver, and plasma compared to control rats. However, administration of CoQ10 significantly reduced the APOP level in these tissues and plasma of CCl_4_ + CoQ10 rats (*p* ≤ 0.01 in kidney and liver; *p* ≤ 0.05 in plasma). Similar effects were also observed in rats supplemented with silymarin. Again, the effects exhibited by CoQ10 and silymarin were statistically similar (Figure 2g–i).

### 3.4. Effect of CoQ10 and Silymarin Supplementation on Antioxidant Enzymes in Kidney, Liver, and Plasma of Ovariectomized Rats

**Figure 3:**
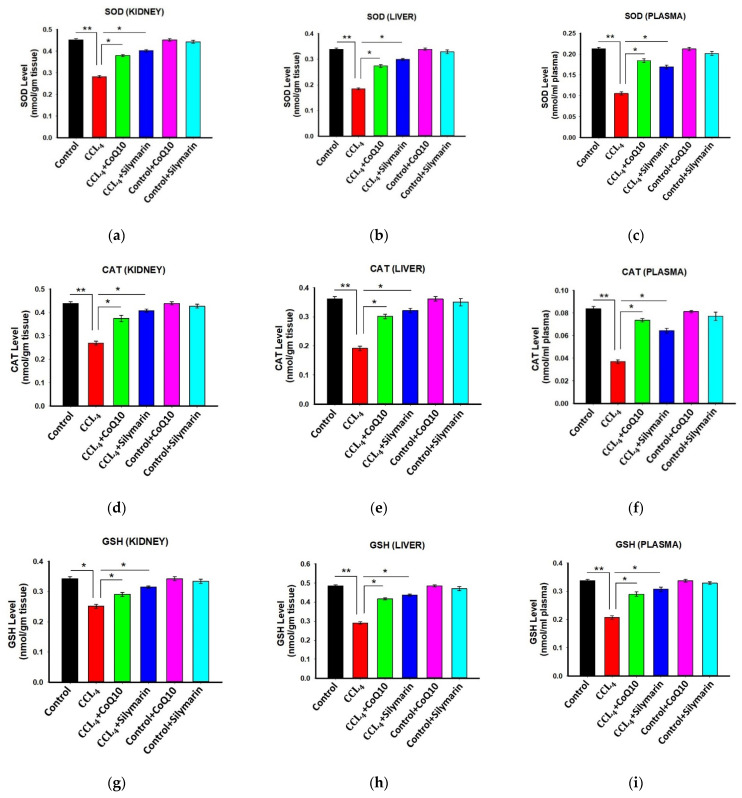
Effect of CoQ10 and silymarin on superoxide dismutase (SOD) (**a**–**c**), catalase (**d**–**f**), and reduced glutathione (GSH) (**g**–**i**) levels in kidney, liver, and plasma of ovariectomized female rats. Data are presented as mean ± SD (n = 6). Statistical analysis involved post-hoc tests. Statistical significance is indicated as * *p* ≤ 0.05 and ** *p* ≤ 0.01.

Next, we investigated the status of antioxidant enzymes in different groups of rats. The results showed that the ovariectomized rats treated with CCl_4_ had significantly lower levels of SOD, CAT, and GSH in kidney, liver, and plasma samples (Figure 3) compared to control rats. However, co-administration of CoQ10 or silymarin reversed all these levels (*p* ≤ 0.05) in a similar pattern. On the other hand, control, control + CoQ10, and control + silymarin rats showed similar levels of SOD, CAT and GSH.

### 3.5. Effect of CoQ10 and Silymarin Supplementation on ALP, AST, and ALT Levels in Plasma

**Figure 4:**
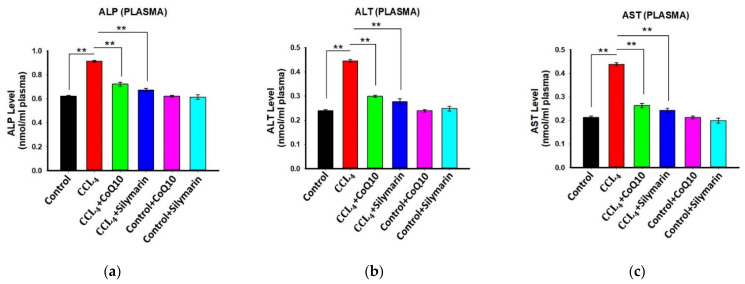
Effect of CoQ10 and silymarin on ALP (**a**), ALT (**b**) and AST (**c**) levels in plasma of ovariectomized female rats. Data are presented as mean ± SD (n = 6). Statistical analysis involved post-hoc tests. Statistical significance is indicated as ** *p* ≤ 0.01.

Administration of CCl_4_ caused significant increase in plasma ALP, AST, and ALT levels in the disease group as compared to the control group. However, supplementation of CoQ10 showed a significant reduction in ALP, AST, and ALT levels in plasma as compared to the disease group (*p* values ≤ 0.05 to ≤ 0.01). Silymarin supplementation also showed a similar effect in reducing plasma levels of ALP, AST, and ALT (Figure 4a–c). We further observed that CoQ10 and silymarin treatment in control rats had no effect on these parameters.

### 3.6. Histopathological Observation

**Figure 5:**
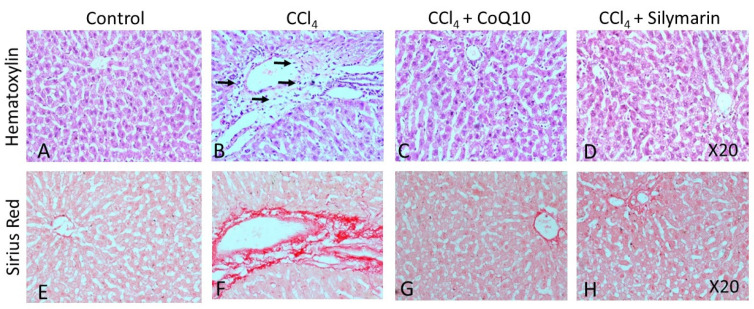
Effect of CCl_4_ and supplementation with CoQ10 and silymarin on liver tissue. Hemtoxylin-eosin staining shows the cellular morphology in control (**A**), CCl_4_ (**B**), CCl_4_ + CoQ10 (**C**), and CCl_4_ + silymarin (**D**) liver of ovariectomized rats. Arrows indicate infiltrating cells. Sirius Red staining depicts fibrosis in control (**E**), CCl_4_ (**F**), CCl_4_ + CoQ10 (**G**), and CCl_4_ + silymarin (**H**) liver of ovariectomized rats. 20× magnification.

**Figure 6:**
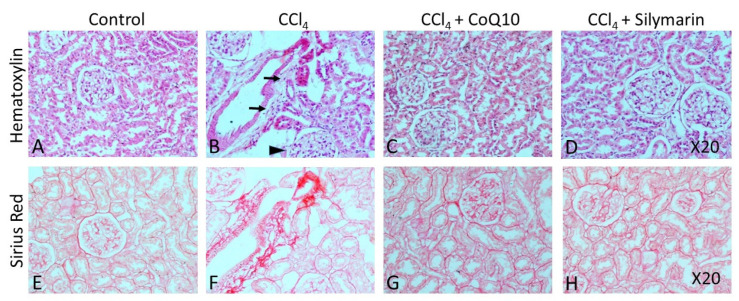
Effect of CCl_4_ and supplementation with CoQ10 and silymarin on kidney tissue. Hemtoxylin-eosin staining shows the cellular morphology in control (**A**), CCl_4_ (**B**), CCl_4_ + CoQ10 (**C**), and CCl_4_ + silymarin (**D**) kidney of ovariectomized rats. Arrows indicate infiltrating cells. Arrowhead depicts deformed glomerular structure. Sirius Red staining depicts fibrosis in control (**E**), CCl_4_ (**F**), CCl_4_ + CoQ10 (**G**), and CCl_4_ + silymarin (**H**) kidney of ovariectomized rats. 20× magnification.

Hematoxylin-eosin (H & E) staining on liver and kidney sections of the control rats revealed a regular morphology in terms of size, shape, and cellular contacts (Figure 5A and Figure 6A, respectively). There was neither infiltration of cells nor tissue necrosis in these tissues. However, CCl_4_-treated rats showed infiltration of inflammatory, mononuclear cells and necrosis in the liver and kidney (Figure 5B and Figure 6B), which were reversed by concurrent supplementation of CoQ10 or silymarin (Figure 5C,D and Figure 6C,D, respectively). The protective effect of CoQ10 and silymarin was comparable with no significant differences. We further stained both kidney and liver tissue sections from all groups of animals using sirius red, which showed the normal distribution of collagen in control rats (Figure 5E and Figure 6E, respectively), while an intense distribution of collagen fibers in the perivascular and inner areas of the liver and kidney was detected in CCl_4_-treated rats (Figure 5F and Figure 6F, respectively). CCl_4_-treated rats also exhibited fibrotic changes, as demonstrated by an increased perivascular deposition of the collagen fibers indicated by sirius red staining. Micro- and macro-vesicular fatty infiltration and cellular infiltration were obvious. Glomerular structure was also deformed in some cases (Figure 5B). However, in sections obtained from rats that received CoQ10 or silymarin concurrently with CCl_4_, there was a significant reduction in the amount of collagen in both liver and kidney with improved tissue architecture (Figure 5G,H and Figure 6G,H, respectively). Cellular arrangement and collagen deposition in the extracellular space were also regular in CoQ10- and silymarin-treated rats.

## 4. Discussion

**Figure 7:**
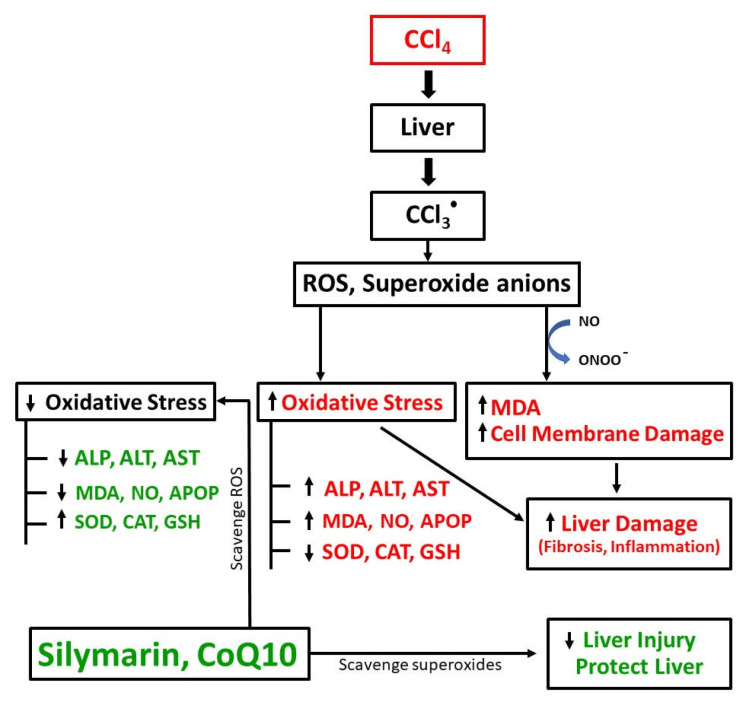
Schematic diagram of CCl_4_-induced pathogenesis of liver damage and protective role of CoQ10 and silymarin.

In this study, we investigated the antioxidant effects of CoQ10 and silymarin and compared their hepatoprotective effects in an experimental ovariectomized rat model in which oxidative stress and cellular damage of kidney and liver were induced by CCl_4_. In the ovariectomized background, the hepatoprotective function of endogenous estrogen was minimized; thus, the exclusive effect of CoQ10 and silymarin was observed. The biochemical analysis showed that CCl_4_ administration significantly impaired liver function biomarkers, while all these parameters were markedly reversed when the animals were supplemented with CoQ10 or silymarin concurrently with CCl_4_. The pathogenesis of liver damage by CCl_4_ and the protective role of CoQ10 and silymarin have been illustrated in Figure 7. At the same time, these biochemical changes were accompanied by histopathological changes, as observed by hematoxylin and sirius red staining. We observed that there were disrupted liver architecture, fibrosis, and accumulation of dark stained nuclei in liver tissue of CCl_4_-treated rats. However, concurrent administration of CoQ10 or silymarin improved the oxidative status, liver enzyme activities, and histopathological changes. Findings from this study suggest that both CoQ10 and silymarin exhibit similar effects in alleviating oxidative stress and protecting liver against oxidative damage.

At a physiological as well as pathological level, reactive oxygen species (ROS) is recurrently produced either due to cellular metabolism or by facing a threat to the homeostasis of the body. The excess free radicals upsurge the oxidative stress markers such as MDA, NO, and APOP [26]. The increased amount of ROS hampers the membrane phospholipids and lowers antioxidant level in respective tissues [27]. In this study, CCl_4_-induced kidney and liver damage increased the stress level, and as a result, stress markers such as MDA, NO, and APOP were found to increase in the disease group, which were again reduced in CoQ10- and silymarin-treated rats. This finding is partially supported by the observation that the circulating concentration of CoQ10 is reduced in patients with chronic kidney disease (CKD), who are in stressed condition, and CoQ10 supplementation minimizes the stress by lowering the MDA level in these patients [10].

The current study revealed that CCl_4_-induced kidney and liver damage was rescued by CoQ10, presumably via its antioxidant activity to scavenge ROS including superoxide anions. Superoxide anions basically bind with NO to form peroxynitrite (ONOO^−^), which causes cellular damage and also promotes lipid peroxidation [28,29,30]. Furthermore, CoQ10 reduced oxidative stress and improved detoxifying enzyme activities as observed in previous studies [31]. Here, we observed that GSH, SOD, and CAT levels were influenced by both silymarin and CoQ10, and these effects were not statistically different. We further observed that CCl_4_ increased liver enzymes (ALT, AST, ALP), which were significantly downregulated by CoQ10 or silymarin. These findings are consistent with previous results demonstrating that the serum ALP, AST, and ALT levels were reduced with subsequent protection against histopathological damage in acetaminophen-, thioacetamide-, CCl_4_-, or hepatic encephalopathy-induced liver injury rat models by CoQ10 or silymarin [32,33,34]. However, the current study differs from several previous studies in that we used an ovariectomized rat model to compare the protective role of CoQ10 and silymarin by observing the changes in oxidative stress markers, antioxidant enzymes, and histopathology simultaneously [34,35,36].

Liver diseases caused by occupational and environmental factors often present a wide clinical spectrum ranging from liver enzyme elevation to liver failure, cirrhosis, and cancer. Hepatocellular necrosis may occur due to exposure to industrial chemicals, primarily solvents [37]. CCl_4_ rats have been proved to be useful experimental models for the study of certain hepatotoxic effects [38,39]. Previous studies have demonstrated that CCl_4_-induced liver fibrosis model mimics several clinical manifestations of human liver fibrosis in different pathological states that consistently produce liver injury with sustained oxidative stress [40]. In the present study, we observed that CoQ10 and silymarin improved liver function. Based on the current findings, CoQ10 and silymarin may be considered for their hepatoprotective potential in the management of chronic liver diseases.

It has been shown that CoQ10, by both direct ROS scavenging and indirect activity via cellular vitamin E and C regeneration and increment of GSH and SOD levels, could act as a potent antioxidant and prevent lipid peroxidation in cellular membranes [41]. We observed that oxidative stress markers MDA, NO, and APOP were significantly increased in kidney tissue of CCl_4_-treated rats; however, concurrent administration of CoQ10 or silymarin markedly reduced these levels and protected kidneys from damage.

Hematoxylin staining has shown the clear morphological abnormalities in both liver and kidney tissues in CCl_4_-tretaed rats. We also observed fibrotic changes in tissue architecture of both liver and kidney. However, concurrent treatment with CoQ10 and silymarin reduced the tissue necrosis, cellular infiltration, and fibrosis, as evidenced from sirius red staining, suggesting that CoQ10 and silymarin protect liver and kidney against oxidative stress.

Previous research showed that CoQ10 is a vital element of the mitochondrial respiratory chain that has gained much attention as a dietary supplement because of its essential function in mitochondrial bioenergetics [42]. Among many biological functions, the hepatoprotective ability of CoQ10 appears as an interesting event, susceptible to counteract several metabolic disorders associated with CCl_4_ toxicity.

From the mechanistic point of view, it has been demonstrated that solubilized CoQ10 impedes transforming growth factor-β1 (TGF-β1) expression in hepatic stellate cells to inhibit fibrosis leading to hepatic damage. Several studies revealed that TGF-β1 downregulates both the expression level and activity of antioxidant enzymes such as glutathione-S-transferase (GST), glutamate cysteine ligase (GCL), SOD, and glutathione peroxidase [43,44,45]. Another study showed that TGF-β1 inhibits the expression of antioxidant enzymes through Smad3/ATF-dependent Nrf2 inactivation [46]. Furthermore, CoQ10 has been found to decrease α-smooth muscle actin (α-SMA) and TGF-β1 expressions in Nrf2 wild-type mouse embryonic fibroblast (MEF) cells, but not in Nrf2-null MEF cells, demonstrating that CoQ10 involves Nrf2 activation to downregulate TGF-β1. Also, Nrf2 overexpression decreased the basal expression of TGF-β1. Therefore, CoQ10-induced Nrf2 activation may suppress TGF-β1 expression and block the fibrosis process, which in turn improves liver health [47]. On the other hand, silymarin has been suggested to block nuclear factor kappa B (NF-kB) by inhibiting interleukin IL-2, IL-4, tumor necrosis factor-α (TNF-α) and interferon-γ (IFN-γ) [48,49] and thus downregulates inflammatory response causing fibrosis. It has also been demonstrated that silymarin acts through lysophosphatidic acid receptor 1 (LPAR1) and TGF-β1 pathways to exert antifibrotic and antiinflammatory effects in hepatic tissue [50,51].

A limitation of this study is that we did not perform experiments to gain insight into the underlying mechanisms of liver damage by CCl_4_ or the molecular pathways of hepatoprotective function of CoQ10 and silymarin. Further studies may be undertaken to address these issues.

## 5. Conclusions

We conclude that this is the first report where ovariectomized female rats were used to investigate the role of CoQ10 and silymarin in absence of estrogen. The results obtained from our study suggest that CoQ10 and silymarin act in a similar pattern in ameliorating the oxidative stress produced by ROS. Also, CoQ10 and silymarin possess comparable antioxidant and hepatoprotective potential, which might be further studied to consider these molecules as supporting therapy in the treatment of chronic diseases such as diabetes, CKD, hypertension, and cancer, where excessive amount of ROS is produced and causes oxidative stress and vital organ injury.

## Data Availability

Not applicable.
